# *rac*-1-(Furan-2-ylmeth­yl)-*N*-nitro-5-(oxolan-2-ylmeth­yl)-1,3,5-triazinan-2-imine

**DOI:** 10.1107/S1600536810036561

**Published:** 2010-09-18

**Authors:** Chuan-Wen Sun, Xu-Bo Ma, Hong-Fei Bu

**Affiliations:** a Department of Chemistry, College of Life and Environmental Science, Shanghai Normal University, Shanghai 200234, People’s Republic of China

## Abstract

In the title compound C_13_H_19_N_5_O_4_, which belongs to the insecticidally active neonicotinoid group of compounds, the triazane ring exhibits a half-chair conformation. The large discrepancy between the two nitro O—N—N bond angles [116.1 (2) and 123.98 (19)°] may be attributed to intra­molecular N—H⋯O hydrogen bonding involving one of the nitro O atoms as the acceptor. The delocalization of the electrons extends as far as the nitro group, forming coplanar π-electron networks. In the crystal, inversion dimers lined by pairs of N—H⋯O hydrogen bonds occur.

## Related literature

For general background to neonicotinoids, see: Kagabu *et al.* (2005 ▶); Peter & Ralf (2008 ▶); Riley & Merz (2007 ▶); Tian *et al.* (2007 ▶); Tomizawa *et al.* (2000 ▶). For the synthesis, see: Zhu *et al.* (2010 ▶).
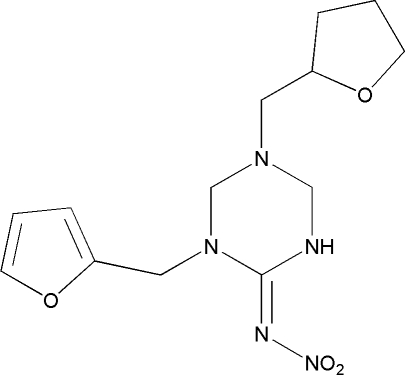


## Experimental

### 

#### Crystal data

C_13_H_19_N_5_O_4_
*M* *_r_* = 309.33
Monoclinic, 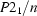

*a* = 11.1898 (12) Å
*b* = 9.262 (1) Å
*c* = 14.4863 (15) Å
β = 99.276 (2)°
*V* = 1481.7 (3) Å^3^
*Z* = 4
Mo *K*α radiation
μ = 0.11 mm^−1^
*T* = 298 K
0.16 × 0.12 × 0.10 mm

#### Data collection

Bruker SMART CCD area-detector diffractometer
9196 measured reflections
2902 independent reflections
2615 reflections with *I* > 2σ(*I*)
*R*_int_ = 0.054

#### Refinement

*R*[*F* ^2^ > 2σ(*F* ^2^)] = 0.066
*wR*(*F* ^2^) = 0.164
*S* = 1.18
2902 reflections
202 parameters
H atoms treated by a mixture of independent and constrained refinement
Δρ_max_ = 0.34 e Å^−3^
Δρ_min_ = −0.21 e Å^−3^

### 

Data collection: *SMART* (Bruker, 2001 ▶); cell refinement: *SAINT* (Bruker, 2001 ▶); data reduction: *SAINT*; program(s) used to solve structure: *SHELXS97* (Sheldrick, 2008 ▶); program(s) used to refine structure: *SHELXL97* (Sheldrick, 2008 ▶); molecular graphics: *SHELXTL* (Sheldrick, 2008 ▶); software used to prepare material for publication: *SHELXTL*.

## Supplementary Material

Crystal structure: contains datablocks I, global. DOI: 10.1107/S1600536810036561/zs2064sup1.cif

Structure factors: contains datablocks I. DOI: 10.1107/S1600536810036561/zs2064Isup2.hkl

Additional supplementary materials:  crystallographic information; 3D view; checkCIF report

## Figures and Tables

**Table 1:** Hydrogen-bond geometry (Å, °)

*D*—H⋯*A*	*D*—H	H⋯*A*	*D*⋯*A*	*D*—H⋯*A*
N2—H2⋯O3	0.82 (3)	1.97 (3)	2.563 (3)	128 (2)
N2—H2⋯O1^i^	0.82 (3)	2.43 (3)	3.035 (3)	132 (2)

Symmetry code: (i) 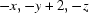.

**Fig. 1.**
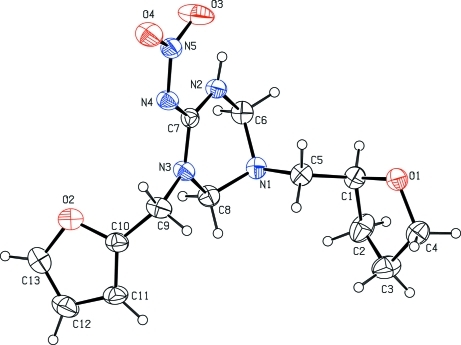
The molecular structure of (I), showing the atom-numbering scheme with non-H atoms shown as 50% probability displacement ellipsoids.

**Fig. 2.**
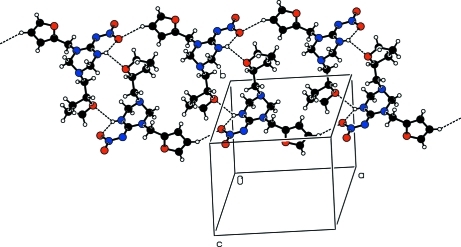
A perspective view of the packing of the title compound (I). Hydrogen bonds are shown as dashed lines.

## supplementary crystallographic information

### Comment

In recent years, the neonicotinoids have been the fastest-growing class of insecticides used in modern crop protection (Tomizawa *et al.*, 2000; Kagabu *et al.*, 2005; Tian *et al.*, 2007; Peter & Ralf, 2008). We report here crystal structure of one of these compounds, C_13_H_19_N_5_O_4_, the title compound (I). In the structure of (I) (Fig. 1), the triazine ring exhibits a half-chair conformation with a dihedral angle of 50.62° between plane A (C8, N3, C7, N2, C6) and plane B (C6, N1, C8). The bond angles C8–N1–C6, N1–C6–N2, C6–N2–C7, N2–C7–N3, C7–N3–C8 and N3–C8–N1 are 108.15 (19), 111.20 (18), 122.69 (19), 118.54 (19), 119.94 (18) and 111.98 (18)° respectively, in turn indicating asymmetry and strong tensility in the 1,3,5-hexahydrotriazine ring. The large discrepancy between the nitro O3–N5–N4 and O4–N5–N4 bond angles [116.1 (2) and 123.98 (19)° respectively] may be attributed to the intramolecular N2–H···O3 hydrogen bond (Table 1). There is also a single intermolecular N–H···O hydrogen bond associated with N2 (Fig. 2).

Interestingly, due to the transfer of the lone-pair of electrons from the hetero-N atoms to the C7═N4 double bond, the C7–N2 and C7–N3 bond lengths (1.327 (3) Å and 1.338 (3) Å), are both remarkably shorter than the pure C–N single bond (1.49 Å), but close to the C═C value (1.33 Å). The delocalization of the electrons extends as far as the electron-withdrawing nitro group, forming a coplanar π-electron network. A six-membered plane C (C7, N4, N5, O3, H2 and N2) is established by the intramolecular N2–H···O3 hydrogen bond. In addition, planes A and C form an enlarged plane D (comprising C6, C8, N3, C7, N2, H2, N4, O3 and O4).

### Experimental

The title compound was prepared by the literature method (Zhu *et al.*, 2010) and was recrystallized from ethanol-water (10:1), giving colorless crystals (yield 79.6%). ^1^HNMR(CDCl_3_, 400 Hz): 9.61 (1*H*, s, NH), 7.37–7.36 (2*H*, d, *J* = 0.8 Hz, furan—H), 6.38–6.34 (3*H*,m,furan—H), 4.49–4.47(6*H*, m, CH_2_—furan, triazine–H), 3.97–3.85 (2*H*, m, CH_2_—tetrahydrofuran), 3.53–3.12 (3*H*, m, tetrahydrofuran—H) 1.86–1.64(4*H*, m, tetrahydrofuran—H); IR(KBr, cm^-1^) 3278(N—H), 1588 (C═N), 1195 (C—O—C), 1060 (C—N), Anal.: calcd. for C_13_H_19_N_5_O_4_: C 50.48, H 6.19, N 22.64%; found, C 51.03, H 6.17, N 22.75%.

### Refinement

H atoms bonded to C were positioned geometrically [C—H = 0.93 Å (aromatic), 0.97 Å (methylene) and 0.98Å (methine)] and refined in riding modes [*U*_iso_(H) = 1.2*U*_eq_(C). H atoms bonded to N were found in Fourier difference maps and refined with the constraints of N—H = 0.82 (3)Å and *U*_iso_(H) = 1.2*U*_eq_(N).

### Crystal data

**Table:**

C_13_H_19_N_5_O_4_	*F*(000) = 656
*M**_r_* = 309.33	*D*_x_ = 1.387 Mg m^−^^3^
Monoclinic, *P*2_1_/*n*	Mo *K*α radiation, λ = 0.71073 Å
Hall symbol: -P 2yn	Cell parameters from 4051 reflections
*a* = 11.1898 (12) Å	θ = 2.5–28.3°
*b* = 9.262 (1) Å	µ = 0.11 mm^−^^1^
*c* = 14.4863 (15) Å	*T* = 298 K
β = 99.276 (2)°	Block, colorless
*V* = 1481.7 (3) Å^3^	0.16 × 0.12 × 0.10 mm
*Z* = 4	

### Data collection

**Table:**

Bruker SMART CCD area-detector diffractometer	2615 reflections with *I* > 2σ(*I*)
Radiation source: fine-focus sealed tube	*R*_int_ = 0.054
graphite	θ_max_ = 26.0°, θ_min_ = 2.1°
φ and ω scans	*h* = −12→13
9196 measured reflections	*k* = −9→11
2902 independent reflections	*l* = −17→17

### Refinement

**Table:**

Refinement on *F*^2^	Primary atom site location: structure-invariant direct methods
Least-squares matrix: full	Secondary atom site location: difference Fourier map
*R*[*F*^2^ > 2σ(*F*^2^)] = 0.066	Hydrogen site location: inferred from neighbouring sites
*wR*(*F*^2^) = 0.164	H atoms treated by a mixture of independent and constrained refinement
*S* = 1.18	*w* = 1/[σ^2^(*F*_o_^2^) + (0.0599*P*)^2^ + 0.7103*P*] where *P* = (*F*_o_^2^ + 2*F*_c_^2^)/3
2902 reflections	(Δ/σ)_max_ < 0.001
202 parameters	Δρ_max_ = 0.34 e Å^−^^3^
0 restraints	Δρ_min_ = −0.21 e Å^−^^3^

### Special details

**Table:**

Geometry. All e.s.d.'s (except the e.s.d. in the dihedral angle between two l.s. planes) are estimated using the full covariance matrix. The cell e.s.d.'s are taken into account individually in the estimation of e.s.d.'s in distances, angles and torsion angles; correlations between e.s.d.'s in cell parameters are only used when they are defined by crystal symmetry. An approximate (isotropic) treatment of cell e.s.d.'s is used for estimating e.s.d.'s involving l.s. planes.
Refinement. Refinement of *F*^2^ against ALL reflections. The weighted *R*-factor *wR* and goodness of fit *S* are based on *F*^2^, conventional *R*-factors *R* are based on *F*, with *F* set to zero for negative *F*^2^. The threshold expression of *F*^2^ > σ(*F*^2^) is used only for calculating *R*-factors(gt) *etc*. and is not relevant to the choice of reflections for refinement. *R*-factors based on *F*^2^ are statistically about twice as large as those based on *F*, and *R*- factors based on ALL data will be even larger.

### Fractional atomic coordinates and isotropic or equivalent isotropic displacement parameters (Å^2^)

**Table:**

	*x*	*y*	*z*	*U*_iso_*/*U*_eq_	
C1	0.1890 (2)	1.0871 (3)	−0.01634 (16)	0.0487 (6)	
H1	0.1262	1.0589	−0.0684	0.058*	
C2	0.2996 (3)	1.1394 (3)	−0.0536 (2)	0.0696 (8)	
H2A	0.3568	1.0614	−0.0561	0.084*	
H2B	0.2777	1.1802	−0.1157	0.084*	
C3	0.3511 (3)	1.2516 (4)	0.0146 (2)	0.0736 (9)	
H3A	0.3924	1.3257	−0.0156	0.088*	
H3B	0.4078	1.2094	0.0651	0.088*	
C4	0.2436 (3)	1.3124 (3)	0.0502 (2)	0.0615 (7)	
H4A	0.2630	1.3307	0.1169	0.074*	
H4B	0.2191	1.4027	0.0188	0.074*	
C5	0.2134 (2)	0.9639 (3)	0.05245 (16)	0.0473 (6)	
H5A	0.2792	0.9902	0.1017	0.057*	
H5B	0.1420	0.9468	0.0809	0.057*	
C6	0.1429 (2)	0.7399 (3)	−0.02488 (15)	0.0482 (6)	
H6A	0.1663	0.6655	−0.0656	0.058*	
H6B	0.0791	0.7970	−0.0608	0.058*	
C7	0.16647 (19)	0.6493 (2)	0.13610 (15)	0.0382 (5)	
C8	0.3341 (2)	0.7465 (3)	0.06578 (16)	0.0476 (6)	
H8A	0.4025	0.8074	0.0900	0.057*	
H8B	0.3630	0.6705	0.0290	0.057*	
C9	0.3679 (2)	0.6694 (3)	0.23428 (16)	0.0532 (6)	
H9A	0.3267	0.6217	0.2798	0.064*	
H9B	0.3899	0.7657	0.2574	0.064*	
C10	0.4795 (2)	0.5882 (3)	0.22556 (16)	0.0482 (6)	
C11	0.5939 (2)	0.6300 (3)	0.23025 (18)	0.0540 (6)	
H11	0.6233	0.7234	0.2408	0.065*	
C12	0.6623 (2)	0.5070 (4)	0.2163 (2)	0.0693 (8)	
H12	0.7454	0.5036	0.2161	0.083*	
C13	0.5869 (3)	0.3982 (4)	0.2035 (3)	0.0808 (10)	
H13	0.6081	0.3034	0.1925	0.097*	
N1	0.24528 (17)	0.8309 (2)	0.00685 (12)	0.0440 (5)	
N2	0.09726 (18)	0.6721 (2)	0.05406 (13)	0.0434 (5)	
H2	0.024 (2)	0.659 (3)	0.0503 (18)	0.052*	
N3	0.28447 (16)	0.6810 (2)	0.14521 (12)	0.0442 (5)	
N4	0.13154 (16)	0.5958 (2)	0.21520 (13)	0.0463 (5)	
N5	0.01557 (17)	0.5609 (2)	0.21385 (14)	0.0486 (5)	
O1	0.14841 (15)	1.20885 (19)	0.03162 (12)	0.0540 (5)	
O2	0.47092 (18)	0.4451 (2)	0.20884 (18)	0.0799 (7)	
O3	−0.06477 (17)	0.5800 (3)	0.14598 (15)	0.0902 (8)	
O4	−0.00984 (17)	0.5099 (2)	0.28658 (14)	0.0709 (6)	

### Atomic displacement parameters (Å^2^)

**Table:**

	*U*^11^	*U*^22^	*U*^33^	*U*^12^	*U*^13^	*U*^23^
C1	0.0558 (14)	0.0487 (14)	0.0407 (12)	−0.0013 (11)	0.0050 (10)	0.0002 (10)
C2	0.092 (2)	0.0565 (17)	0.0706 (18)	−0.0087 (16)	0.0424 (17)	0.0028 (14)
C3	0.0628 (17)	0.078 (2)	0.084 (2)	−0.0169 (16)	0.0242 (16)	−0.0094 (17)
C4	0.0651 (17)	0.0516 (16)	0.0669 (17)	−0.0046 (13)	0.0075 (13)	−0.0092 (13)
C5	0.0543 (14)	0.0514 (14)	0.0384 (11)	−0.0023 (11)	0.0141 (10)	0.0002 (10)
C6	0.0558 (14)	0.0530 (14)	0.0361 (11)	0.0010 (11)	0.0086 (10)	−0.0008 (10)
C7	0.0385 (11)	0.0360 (11)	0.0409 (11)	0.0031 (9)	0.0088 (9)	−0.0005 (9)
C8	0.0448 (12)	0.0543 (14)	0.0470 (12)	0.0011 (11)	0.0175 (10)	0.0064 (11)
C9	0.0432 (13)	0.0758 (18)	0.0407 (12)	−0.0032 (12)	0.0072 (10)	0.0061 (12)
C10	0.0418 (12)	0.0613 (16)	0.0393 (11)	−0.0063 (11)	0.0004 (9)	0.0113 (11)
C11	0.0406 (12)	0.0648 (16)	0.0580 (14)	−0.0129 (12)	0.0121 (11)	0.0088 (12)
C12	0.0416 (14)	0.093 (2)	0.0730 (18)	0.0042 (15)	0.0090 (13)	0.0168 (17)
C13	0.0614 (18)	0.069 (2)	0.108 (3)	0.0143 (17)	0.0027 (17)	0.0073 (19)
N1	0.0493 (11)	0.0454 (11)	0.0393 (9)	0.0019 (9)	0.0125 (8)	0.0025 (8)
N2	0.0386 (10)	0.0493 (12)	0.0416 (10)	−0.0025 (9)	0.0046 (8)	0.0033 (8)
N3	0.0370 (10)	0.0562 (12)	0.0403 (10)	0.0008 (8)	0.0091 (8)	0.0102 (8)
N4	0.0369 (10)	0.0568 (12)	0.0465 (10)	−0.0022 (9)	0.0099 (8)	0.0112 (9)
N5	0.0423 (11)	0.0519 (12)	0.0523 (12)	−0.0010 (9)	0.0101 (9)	0.0095 (9)
O1	0.0495 (10)	0.0493 (10)	0.0636 (11)	0.0033 (8)	0.0104 (8)	−0.0040 (8)
O2	0.0524 (12)	0.0665 (14)	0.1163 (19)	−0.0084 (10)	−0.0001 (11)	0.0061 (12)
O3	0.0461 (11)	0.154 (2)	0.0675 (13)	−0.0258 (13)	0.0006 (10)	0.0314 (14)
O4	0.0549 (11)	0.0947 (16)	0.0677 (12)	−0.0047 (10)	0.0235 (9)	0.0345 (11)

### Geometric parameters (Å, °)

**Table:**

C1—O1	1.436 (3)	C7—N4	1.362 (3)
C1—C2	1.508 (4)	C8—N1	1.433 (3)
C1—C5	1.510 (3)	C8—N3	1.485 (3)
C1—H1	0.9800	C8—H8A	0.9700
C2—C3	1.485 (4)	C8—H8B	0.9700
C2—H2A	0.9700	C9—N3	1.469 (3)
C2—H2B	0.9700	C9—C10	1.481 (3)
C3—C4	1.494 (4)	C9—H9A	0.9700
C3—H3A	0.9700	C9—H9B	0.9700
C3—H3B	0.9700	C10—C11	1.329 (3)
C4—O1	1.426 (3)	C10—O2	1.348 (3)
C4—H4A	0.9700	C11—C12	1.405 (4)
C4—H4B	0.9700	C11—H11	0.9300
C5—N1	1.469 (3)	C12—C13	1.309 (4)
C5—H5A	0.9700	C12—H12	0.9300
C5—H5B	0.9700	C13—O2	1.382 (4)
C6—N1	1.436 (3)	C13—H13	0.9300
C6—N2	1.467 (3)	N2—H2	0.82 (3)
C6—H6A	0.9700	N4—N5	1.335 (3)
C6—H6B	0.9700	N5—O4	1.229 (3)
C7—N2	1.327 (3)	N5—O3	1.233 (3)
C7—N3	1.338 (3)		
			
O1—C1—C2	105.2 (2)	N1—C8—N3	111.98 (18)
O1—C1—C5	108.15 (18)	N1—C8—H8A	109.2
C2—C1—C5	114.1 (2)	N3—C8—H8A	109.2
O1—C1—H1	109.7	N1—C8—H8B	109.2
C2—C1—H1	109.7	N3—C8—H8B	109.2
C5—C1—H1	109.7	H8A—C8—H8B	107.9
C3—C2—C1	103.8 (2)	N3—C9—C10	112.8 (2)
C3—C2—H2A	111.0	N3—C9—H9A	109.0
C1—C2—H2A	111.0	C10—C9—H9A	109.0
C3—C2—H2B	111.0	N3—C9—H9B	109.0
C1—C2—H2B	111.0	C10—C9—H9B	109.0
H2A—C2—H2B	109.0	H9A—C9—H9B	107.8
C2—C3—C4	104.2 (2)	C11—C10—O2	109.6 (2)
C2—C3—H3A	110.9	C11—C10—C9	131.8 (3)
C4—C3—H3A	110.9	O2—C10—C9	118.6 (2)
C2—C3—H3B	110.9	C10—C11—C12	107.4 (3)
C4—C3—H3B	110.9	C10—C11—H11	126.3
H3A—C3—H3B	108.9	C12—C11—H11	126.3
O1—C4—C3	107.4 (2)	C13—C12—C11	106.9 (3)
O1—C4—H4A	110.2	C13—C12—H12	126.6
C3—C4—H4A	110.2	C11—C12—H12	126.6
O1—C4—H4B	110.2	C12—C13—O2	109.9 (3)
C3—C4—H4B	110.2	C12—C13—H13	125.1
H4A—C4—H4B	108.5	O2—C13—H13	125.1
N1—C5—C1	111.59 (18)	C8—N1—C6	108.15 (19)
N1—C5—H5A	109.3	C8—N1—C5	112.63 (19)
C1—C5—H5A	109.3	C6—N1—C5	113.40 (19)
N1—C5—H5B	109.3	C7—N2—C6	122.69 (19)
C1—C5—H5B	109.3	C7—N2—H2	117.9 (18)
H5A—C5—H5B	108.0	C6—N2—H2	118.7 (18)
N1—C6—N2	111.20 (18)	C7—N3—C9	123.22 (18)
N1—C6—H6A	109.4	C7—N3—C8	119.94 (18)
N2—C6—H6A	109.4	C9—N3—C8	116.49 (18)
N1—C6—H6B	109.4	N5—N4—C7	119.09 (19)
N2—C6—H6B	109.4	O4—N5—O3	119.9 (2)
H6A—C6—H6B	108.0	O4—N5—N4	116.1 (2)
N2—C7—N3	118.54 (19)	O3—N5—N4	123.98 (19)
N2—C7—N4	127.3 (2)	C4—O1—C1	109.54 (19)
N3—C7—N4	114.15 (19)	C10—O2—C13	106.2 (2)
			
O1—C1—C2—C3	29.5 (3)	N1—C6—N2—C7	−25.4 (3)
C5—C1—C2—C3	−88.8 (3)	N2—C7—N3—C9	175.7 (2)
C1—C2—C3—C4	−30.1 (3)	N4—C7—N3—C9	−4.3 (3)
C2—C3—C4—O1	20.2 (3)	N2—C7—N3—C8	2.8 (3)
O1—C1—C5—N1	175.01 (18)	N4—C7—N3—C8	−177.2 (2)
C2—C1—C5—N1	−68.3 (3)	C10—C9—N3—C7	130.5 (2)
N3—C9—C10—C11	110.1 (3)	C10—C9—N3—C8	−56.4 (3)
N3—C9—C10—O2	−69.1 (3)	N1—C8—N3—C7	28.7 (3)
O2—C10—C11—C12	−0.3 (3)	N1—C8—N3—C9	−144.7 (2)
C9—C10—C11—C12	−179.6 (2)	N2—C7—N4—N5	−0.1 (4)
C10—C11—C12—C13	0.3 (3)	N3—C7—N4—N5	179.9 (2)
C11—C12—C13—O2	−0.2 (4)	C7—N4—N5—O4	178.3 (2)
N3—C8—N1—C6	−56.5 (3)	C7—N4—N5—O3	−3.5 (4)
N3—C8—N1—C5	69.6 (2)	C3—C4—O1—C1	−1.6 (3)
N2—C6—N1—C8	54.5 (2)	C2—C1—O1—C4	−17.4 (3)
N2—C6—N1—C5	−71.2 (2)	C5—C1—O1—C4	104.8 (2)
C1—C5—N1—C8	144.4 (2)	C11—C10—O2—C13	0.2 (3)
C1—C5—N1—C6	−92.4 (2)	C9—C10—O2—C13	179.6 (2)
N3—C7—N2—C6	−4.4 (3)	C12—C13—O2—C10	0.0 (4)
N4—C7—N2—C6	175.6 (2)		

### Hydrogen-bond geometry (Å, °)

**Table:**

*D*—H···*A*	*D*—H	H···*A*	*D*···*A*	*D*—H···*A*
N2—H2···O3	0.82 (3)	1.97 (3)	2.563 (3)	128 (2)
N2—H2···O1^i^	0.82 (3)	2.43 (3)	3.035 (3)	132 (2)

Symmetry codes: (i) −*x*, −*y*+2, −*z*.
